# Femoral Vein Pulsatility and Neurocognitive Disorder in Cardiac Surgery

**DOI:** 10.1016/j.cjco.2024.11.002

**Published:** 2024-11-08

**Authors:** Ali Hammoud, Tanya Mailhot, Melissa Parent, Karel Huard, Olivier Lachance, Patrick Tawil, Alexander Calderone, Sylvie Levesque, Stéphanie Jarry, William Beaubien-Souligny, Étienne J. Couture, André Y. Denault

**Affiliations:** a Intensive Care Unit, Montreal Heart Institute, Montréal, Québec, Canada; b Montreal Heart Institute, Université de Montréal, Montréal, Québec, Canada; c Université de Montréal, Montréal, Québec, Canada; d Department of Medicine, Jewish General Hospital, McGill University Health Centre, Montréal, Québec, Canada; e Montreal Health Innovations Coordinating Centre, Montreal Heart Institute, Montréal, Québec, Canada; f Department of Anesthesiology, Montreal Heart Institute, Université de Montréal, Montréal, Québec, Canada; g Division of Nephrology, Centre Hospitalier de l'Université de Montréal, Montréal, Québec, Canada; h Department of Anesthesiology and Division of Intensive Care Medicine, Institut Universitaire de Cardiologie et de Pneumologie de Québec, Université Laval, Québec City, Québec, Canada

## Abstract

**Background:**

Neurocognitive disorder and venous congestion are related in cardiac surgery. Femoral vein (FV) Doppler allows noninvasive assessment of venous congestion. This study aims to associate postoperative neurocognitive disorder in cardiac surgery with FV pulsatility.

**Method:**

A secondary analysis was conducted using data from retrospective and prospective cohorts. FV Doppler and neurocognitive disorder were measured upon admission to the intensive care unit (ICU) after surgery. An index of FV pulsatility of ≥ 50% was considered abnormal. The presence of neurocognitive disorder was assessed using the Intensive Care Delirium Score Checklist (ICDSC). Descriptive statistical analyses and logistic regression were used to test the association between FV Doppler pulsatility and neurocognitive disorder.

**Results:**

A total of 273 patients from both cohorts were analyzed, 155 (57%) patients had FV pulsatility indexes ≥ 50%. Abnormal pulsatile Doppler was associated with the presence of neurocognitive disorder compared with normal FV Doppler profile (57 vs 43%, odds ratio [OR], 1.73; 95% confidence interval [CI], 1.06-2.81). FV pulsatility was also associated with age, higher rate of stroke, prolongation of intubation duration and a longer ICU stay.

**Conclusions:**

FV pulsatility is associated with the presence of neurocognitive disorder and postoperative complications in cardiac surgery. The FV Doppler technique is simple and fast, offering the potential to anticipate complications related to venous congestion, such as delirium. Future multicentre studies with larger samples will be necessary to consolidate these findings.

**Clinical Registration Number:**

NCT04092855 and NCT05038267.

## Graphical abstract

**Figure:**
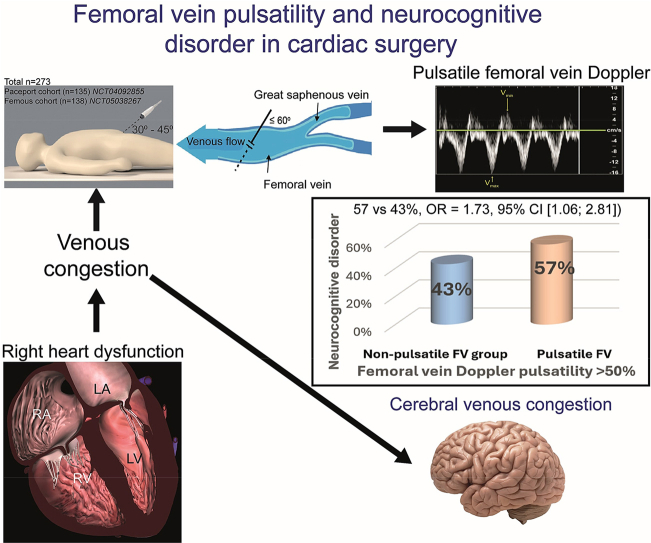

## Résumé

**Contexte:**

Les troubles neurocognitifs et la congestion veineuse sont liés lors des chirurgies cardiaques. Le Doppler de la veine fémorale (VF) permet une évaluation non invasive de la congestion veineuse. Cette étude vise à associer les troubles neurocognitifs postopératoires en chirurgie cardiaque à la pulsatilité de la VF.

**Méthode:**

Une analyse secondaire a été réalisée à partir de données provenant de cohortes rétrospectives et prospectives. Le Doppler de la VF et les troubles neurocognitifs ont été mesurés lors de l'admission à l'unité de soins intensifs (USI) après la chirurgie. Un indice de pulsatilité de la VF ≥ 50 % a été considéré comme anormal. La présence de troubles neurocognitifs a été évaluée à l'aide de la liste de contrôle du score de délirium aux soins intensifs. Des analyses statistiques descriptives et des régressions logistiques ont été utilisées pour tester l'association entre la pulsatilité du Doppler de la VF et les troubles neurocognitifs.

**Résultats:**

Au total, 273 patients des deux cohortes ont été inclus, 155 (57 %) patients présentaient des indices de pulsatilité de la VF ≥ 50 %. Un Doppler pulsatile anormal était associé à la présence de troubles neurocognitifs par rapport à un profil Doppler de la VF normal (57 contre 43 %, rapport des cotes, 1,73; intervalle de confiance à 95 %, 1,06 à 2,81). La pulsatilité de la VF était également associée à l'âge, à un taux plus élevé d'accidents vasculaires cérébraux, à une durée d'intubation plus longue et à un séjour plus long en USI.

**Conclusions:**

La pulsatilité de la VF est associée à la présence de troubles neurocognitifs et de complications postopératoires en chirurgie cardiaque. La technique du Doppler de la VF est simple et rapide, offrant la possibilité d'anticiper les complications liées à la congestion veineuse, telles que le délirium. De futures études multicentriques avec un échantillonnage plus important seront nécessaires pour consolider ces résultats.

Postoperative cognitive impairment in cardiac surgery affects up to 70% of patients admitted to the intensive care unit (ICU).^1^ Among this cognitive impairment, delirium and subsyndromal delirium (SSD) represent the largest proportion of such issues. According to the Diagnostic and Statistical Manual of Mental Disorders (DSM-V), delirium involves acute alterations in attention and consciousness.^2^ Individuals experiencing delirium may also have hallucinations, memory and speech disturbances, and fluctuations in sleep patterns. On the other hand, SSD is a milder form of delirium that involves some cognitive alterations in the postoperative period but does not meet all the diagnostic criteria of the DSM-V.^3^ Cognitive impairment following cardiac surgery complicates recovery and increases hospital length of stay and the risk of mortality.^4^

The mechanism of the pathogenesis of delirium and SSD remains unclear. The most probable explanation in the postoperative period of cardiac surgery is the increase in inflammatory activity and the immunohormonal response caused by surgical stress.^5^ This response causes alterations in microcirculation and increases the permeability of the blood–brain barrier, leading to neurotransmission impairment and disruption of neuronal function.^5^ In addition to this inflammatory state, some studies suggest the significant role of fluid overload leading to venous congestion in the development of postoperative delirium in cardiac surgery.^6^ The hypothesis is that edema in cerebral structures accompanying systemic venous congestion may result in brain dysfunction through alterations in perfusion and cerebral microcirculation.^7^ The diagnosis of systemic venous congestion can be made using splanchnic venous Doppler.^8^ Indeed, portal vein pulsatility has been associated with delirium after cardiac surgery.^9^ Femoral venous (FV) Doppler interrogation correlates with portal venous Doppler and may represent a simpler alternative to identify the presence of venous congestion.^10^ A normal FV Doppler signal is continuous but becomes pulsatile in the presence of venous congestion.^10^^,^^11^ Therefore, the primary objective of this study is to assess the presence of an association between postoperative neurocognitive disorder in cardiac surgery and abnormal FV Doppler. The secondary objective is to compare postoperative complications according to the presence of abnormal FV Doppler or not.

## Methodology

This study included data from 2 cohorts of patients who underwent cardiac surgery. Data from the first cohort were retrospectively collected between May 2019, and March 2022, from consecutive patients undergoing cardiac surgery (PACEPORT cohort: clinicaltrials.org identifier: NCT04092855).^12^ Data from the second cohort were prospectively collected between August 2021 and June 2022 from consecutive patients undergoing cardiac surgery (FEMOUS cohort: clinicaltrials.org identifier: NCT05038267). Both studies were approved by the Ethics Committee of the Montreal Heart Institute (2019-2527 and 2021-2963). Written informed consent was obtained from all participants in both cohorts.

### Participants

Both cohorts consist of adult patients, aged 18 and older, who underwent elective cardiac surgery and had an ultrasound measurement of FV Doppler. In the PACEPORT study, patients with concurrent conditions such as pericardial constriction, congenital heart disease, severe valvular regurgitation, right ventricular systolic dysfunction, or right ventricular infarction were excluded. In the FEMOUS cohort, patients with liver cirrhosis, pregnant women, those with preoperative intra-aortic balloon, femoral vein thrombosis, or those with inaccessible bilateral FV were specifically excluded. No patients with postoperative right ventricular dysfunction were excluded. For this secondary analysis, we included all patients from both cohorts for whom all FV Doppler data and assessment of the presence of neurocognitive disorder were available.

### FV Doppler assessment

FV Doppler ultrasound was conducted using a high-frequency linear probe at ICU admission within the first 2 hours. Patients were positioned in a dorsal decubitus up to 10°. Measurements were taken on both right and left FV in both transversal (short axis) and longitudinal (long axis) planes. Longitudinal measurements were taken with an angle adjustment of less than 60°. Pulsatility index (PI) was defined as the maximal velocity, minimal velocity divided by the maximal velocity value $\left(\frac{Vmax-Vmin}{Vmax}\right)$. Abnormal PI was defined as ≥ 50 %.^13^ Velocities were measured at the end of expiration (Supplemental Figs, S1 and S2).

Measurements of FV Doppler were obtained at the time of the patient's admission to the ICU following surgery. All ultrasound measurements were performed by the research team trained and supervised by a physician certified in ICU ultrasound (A.Y.D.).

### Assessment of neurocognitive disorder

The presence of neurocognitive disorder was assessed using the Intensive Care Delirium Score Checklist (ICDSC) tool, an 8-item instrument for delirium detection. A score of 4 or more out of 8 indicates the presence of delirium, whereas a score of 1 to 3 indicates the presence of SSD.^14^ We considered the presence of neurocognitive disorder if the score were equal to 1 or more. This tool is validated for the critically ill population and has a sensitivity of 81% and a specificity of 87.7% for detection of delirium compared with a formal psychiatric evaluation.^15^ In the PACEPORT and FEMOUS cohorts, the ICDSC score were independently assessed by ICU nurses at least 3 times a day and recorded on the nursing observation sheet. Nurses were not informed of the FV pulsatility score. The highest score recorded since patient arrival in the ICU unit after surgery until 48 hours postoperatively was noted. If the score was not recorded in the specific assessment grid, it was considered equal to 0, unless a medical or nursing note mentioned the presence of delirium or neurocognitive disorder, in which case it was considered equal to 1. Patients whose cognitive status could not be assessed, for example, because of prolonged sedation of more than 48 hours, as well as those who had pre-existing neurocognitive disorders were excluded from the final analysis.

### Other clinical data

The demographic and clinical characteristics of collected patient data included age, gender, body mass index (BMI), alcohol and tobacco consumption, preoperative risk assessed by the European System for Cardiac Operative Risk Evaluation (EuroSCORE II), left ventricular function, renal function, diabetes, and the presence of chronic lung disease.

Perioperative patient characteristics and complications included the type of surgery (coronary revascularization, valvular surgeries, aortic surgery, and other types of cardiac surgeries), duration of cardiopulmonary bypass (CPB) in hours, CPB weaning status, postoperative complications (major bleeding,^16^ acute renal failure,^17^ duration of dialysis in days, stroke, reintervention, duration of ventilation in hours, duration of vasopressors in hours, duration of stay in the ICU in hours, total duration of hospitalization in days, and the total time of persistent organ dysfunction [TPOD] in hours) (Definitions in Supplemental Table S1).

### Statistical analyses

Descriptive statistics for discrete and categorical variables are presented as proportions and percentages. Continuous variables are presented as median and interquartile range (IQR) or mean and standard deviation according to their distribution.

To address the primary objective, logistic regression analysis was conducted to control for other variables associated with FV Doppler or neurocognitive disorder. The dependent variable was the presence of neurocognitive disorder, and the independent variables (main and control) were the Doppler ultrasound measurement of the FV (abnormal if PI ≥ 50% vs normal if PI < 50%), age, and severity of hemodynamic instability upon admission to the ICU, measured by the duration of vasopressor use.

To address the secondary objective, χ^2^ tests were performed for all categorical data. Comparison among group variables was conducted using a Student's *t*-test for means of normally distributed variables or with a nonparametric test (Mann-Whitney U test for 2 samples) for medians of non-normally distributed variables. The statistical tests used are 2-sided with a significance threshold of 0.05. The statistical analysis was conducted using the SPSS software (IBM SPSS Software Statistics, version 27, Armonk, NY).

## Results

In the PACEPORT cohort we included 135 out of 190 patients, whereas in the FEMOUS cohort, 138 of 150 recruited patients were included, for a total of 273 patients (Supplemental Fig. S3). In the FEMOUS cohort, adequate femoral venous Doppler was obtained in 147 of 150 (98.0%) on admission to ICU and in 149 of 150 (99.3%) on postoperative day 1.

Among the 273 analyzed patients, 155 (57%) had pulsatile FV Doppler compared with 118 (43%) with nonpulsatile Doppler upon admission to ICU. The FV pulsatile group exhibited an older age distribution, with a median age of 70 years (IQR: 63-76), compared with 68 years (IQR: 60-73) in the nonpulsatile group (*P* = 0.033). No other statistical difference was found between the 2 groups in terms of EuroSCORE II, gender, alcohol consumption, smoking, BMI, preoperative creatinine, diabetes, and chronic lung disease. In addition, no significant difference was observed between the groups concerning the type of surgery, duration, and CPB separation classification (Table 1). In terms of postoperative complications, the pulsatile FV Doppler group had longer mechanical ventilation duration (3 hours (IQR: 2.3-4.5) vs 2.8 (IQR: 2-4), *P* = 0.023), longer stay in the ICU (47 hours (IQR: 23-72) vs 28 hours (IQR: 22-57), *P* = 0.045). All 5 strokes and the 2 deaths were in the FV pulsatile Doppler group. There were no significant differences between the groups for major bleeding, acute kidney injury, surgical reintervention, total hospitalization duration, and TPOD (Table 2).

**Table 1:** Preoperative clinical characteristics of patients according to their FV Doppler profile

Variables	Entire cohort N = 273	Nonpulsatile FV N = 118	Pulsatile FV N = 155	*P* value
Age (years)	69 (62; 75)	68 (60; 73)	70 (63; 76)	0.033
Male gender	208 (76)	94 (80)	114 (74)	0.240
BMI (kg/m^2^)	28 (25; 32)	28 (25; 33)	27 (24; 31)	0.075
Alcohol use disorder	29 (21)	15 (22)	14 (20)	0.700
Active smoker	19 (13.7)	13 (19)	6 (8)	0.062
EuroSCORE II	1.60 (0.95; 2.81)	1.41 (0.83; 2.43)	1.73 (1.04; 2.95)	0.105
LV function				0.337
Good (> 50%)	201 (73)	91 (77)	110 (71)	
Moderate (40%-49%)	57 (21)	22 (19)	35 (23)	
Poor (30%-39%)	14 (5)	4 (3)	10 (6)	
Severe (< 30%)	1 (0.4)	1 (0.8)	0 (0)	
Preoperative creatinine (mmol/L)	83 (73; 96)	81 (71; 95)	84 (75; 96)	0.103
Diabetes	88 (32)	33 (30)	55 (35)	0.188
Chronic lung disease	46 (17)	20 (17)	26 (17)	0.969
History of cardiac surgery	19 (7)	9 (8)	10 (6)	0.705
Type of surgery				0.141
Coronary revascularization	132 (48)	65 (55)	67 (43)	
Valvular	62 (23)	22 (19)	40 (26)	
Coronary revascularization and valvular	45 (17)	15 (13)	30 (19)	
Other types of cardiac surgery	34 (12)	16 (13)	18 (12)	
CPB duration (min)	95.49 ± 46.42	94.33 ± 46.64	93.14 ± 41.74	0.832
CPB separation classification	N = 265	N = 114	N = 151	
Easy	192 (72)	83 (73)	109 (72)	
Difficult	58 (22)	24 (21)	34 (23)	
Complex	15 (6)	7 (6)	8 (5)	

Variable definitions can be found in Supplemental Table S1.

Values are presented as median (interquartile range), mean ± SD, or n (%).

σ, standard deviation; BMI, body mass index; CPB, cardiopulmonary bypass; FV, femoral vein; IQR, interquartile range; LV, left ventricle (see definition of LV function in Supplemental Table S1).

European System for Cardiac Operative Risk Evaluation (EuroSCORE II) online calculator (http://www.euroscore.org/calc.html).

**Table 2:** Postoperative complications according to FV Doppler profile

Variables	Entire cohort N = 273	Nonpulsatile FV N = 118	Pulsatile FV N = 155	*P* value
Major bleeding	9 (3)	3 (3)	6 (4)	0.542
Acute kidney injury				0.129
Absence	207 (76)	92 (78)	115 (74)	
Stage 1	48 (18)	18 (15)	30 (19)	
Stage 2	9 (3)	2 (2)	7 (5)	
Stage 3	8 (3)	6 (5)	3 (2)	
Stroke	5 (1.8)	0 (0)	5 (3)	0.049
Surgical reintervention	11 (4)	5 (4)	6 (4)	0.879
ICU mortality	2 (0.7)	0 (0)	2 (1)	0.216
Mechanical ventilation (hours)	3 (2; 4.46)	2.83 (2; 4)	3 (2.3; 4.5)	0.023
Vasopressor use (hours)	11 (1; 33.5)	9 (1; 29)	9 (2; 33)	0.450
ICU length of stay (hours)	45 (23; 72)	28 (22; 56.75)	47 (23; 72)	0.045
Hospital length of stay (days)	6 (5; 7)	6 (5; 7)	6 (5; 7)	0.550
TPOD (hours)	11 (3; 32)	8 (3; 22)	10 (3; 34)	0.205

Definition of these variables is available in Supplemental Table S1.

Values are presented as median (interquartile range), mean ± SD, or n (%).

CPB, cardiopulmonary bypass; FV, femoral venous; ICU, intensive care unit; IQR, interquartile range; TPOD, time of persistent organ dysfunction.

The neurocognitive disorder was more frequent in the FV pulsatile Doppler group. A total of 87 patients (57%) experienced a cognitive complication in the first 48 hours postoperatively in the FV pulsatile group, compared with 51 patients (43%) in the FV nonpulsatile group (*P* = 0.026) (Table 3). After multivariable adjustment, an independent association between pulsatile FV Doppler signal and the presence of postoperative neurocognitive disorder was observed (OR, 1.78; IQR: 1.009-3.136; *P* = 0.046) (Table 4).

**Table 3:** Distribution of patients according to ultrasound data (descriptive and proportion) and neurocognitive disorder

	Total N = 271∗	Nonpulsatile FV N = 118	Pulsatile FV N = 153	*P* value
Neurocognitive disorder, n (%)	138 (51%)	51 (43%)	87 (57%)	0.026

FV, femoral vein.

∗ N = 271 out of a total of 273 FV Doppler examinations were performed, as we do not have Intensive Care Delirium Score Checklist data for the 2 deceased patients.

**Table 4:** Logistic regression model for postoperative neurocognitive disorder

Parameters	B ± ES	Exp B (OR)	*P* value	CI 95%
Age	0.041 ± 0.015	1.047	0.004	1.013-1.072
Vasopressors duration	0.013 ± 0.006	1.013	0.026	1.002-1.025
Pulsatile FV Doppler ≥ 50%	0.576 ± 0.289	1.779	0.046	1.009-3.136

B, beta coefficient; CI, confidence interval; ES, effect size; Exp B, exponential B; FV, femoral vein; OR, odds ratio.

## Discussion

This cohort study supports an association between abnormal FV Doppler, neurocognitive disorder, and postoperative complications in cardiac surgical patients. This association aligns with previous studies that have also noted a correlation with other signs of venous congestion and cognitive dysfunction, postoperative complications, and mortality.^9^^,^^18^^,^^19^ However, the primary advantage of FV Doppler measurement lies in its simplicity of acquisition and very high success rate.

We observed that patients with pulsatile FV Doppler ultrasound results were older, more frequently experience postcardiac surgery strokes, and had prolonged intubation and ICU stays compared with those with nonpulsatile FV Doppler ultrasound. Indeed, when considering age and vasopressor use, regression analysis showed a statistically significant association between FV Doppler pulsatility and the presence of neurocognitive disorder. This suggests that the degree of venous congestion secondary to abnormally elevated right atrial pressures, leading to abnormal FV Doppler signals, may represent an additional factor associated with postoperative neurocognitive disorder in cardiac surgery patients or congestive encephalopathy.^20^ This mechanism of peripheral and consequently cerebral venous congestion is supported by a growing body of literature on blood–brain barrier impairment observed in the presence of cerebral venous hypertension as previously reported.^21^^,^^22^ Our results are also consistent with other studies in cardiac surgery patients, highlighting the associations among venous congestion, fluid overload, and the risk of delirium and cognitive disorders.^6^^,^^20^ This study is the first to demonstrate in a large cohort of postoperative cardiac surgical patients a potential association between abnormal FV pulsatility Doppler and the presence of postoperative neurocognitive disorder.

The association between abnormal pulsatile FV Doppler, elevated right atrial pressure, and right ventricular dysfunction (RVD) is well documented in the literature.^10^^,^^11^^,^^23^ RVD leads to increased filling pressures and systemic venous congestion, along with brain desaturation using near-infrared spectroscopy^24^ and abnormal electrical brain activity, as recently described by Jarry et al.^25^ Furthermore, RVD is associated with complications such as increased mortality and prolonged hospitalization.^26^ In this context, it is not surprising to observe an associations among pulsatile FV Doppler and longer duration of intubation, ICU stay, and neurocognitive disorder.

### Limitations

Our observations result from the secondary objectives of 2 prospective studies. Consequently, the number of patients was predetermined based on the primary objectives of these 2 studies. This study serves as a preliminary step to power a subsequent confirmatory study adequately. The detection of neurocognitive disorder requires specialized tests for cognitive anomalies, which were not available. We relied on the values of the ICDSC, a validated tool used for screening of delirium, and evaluation was carried out by the ICU nurses, independently, ensuring they were unaware of the results of the FV Doppler ultrasound. Although neurocognitive disorder can occur up to 7 days after surgery, ICDSC data were only collected for 48 hours after surgery. Consequently, delirium that would have emerged later could have been missed. The decision to score the ICDSC as positive or negative, rather than retaining the actual score, was made to simplify the evaluation for practical reasons. Keeping the actual score would have complicated the diagnosis of delirium and SSD, given the broader definition of neurocognitive disorder.

Ideally, to validate our hypothesis that abnormal FV Doppler is associated with congestive delirium, cerebral measures of venous congestion or cerebral biomarkers would have been necessary. For example, S100B could be influenced by any disruption of the blood–brain barrier in case of fluid overload.^27^ Newer brain saturation monitors that allow detection of venous congestion were not available at the time of the study.^28^ The link with venous congestion, RVD, and stroke could be the result of air emboli that can be quantified using transcranial Doppler and has been related to postoperative outcome.^29^ However, transcranial Doppler monitoring was used only in a subset of patients. Finally, additional work will be needed to demonstrate whether the normalization of abnormal FV Doppler signals can reduce the incidence and duration of delirium or neurocognitive disorder after cardiac surgery.

## Conclusions

An association was observed between abnormal pulsatile FV Doppler and postcardiac surgery neurocognitive disorder. The FV Doppler technique is simple and quick. It could help anticipate complications related to venous congestion, including neurocognitive disorder. FV Doppler interrogation could also provide an alternative therapeutic target to improve these conditions as previously reported.^30^ Multicentric future studies with larger samples will be necessary to consolidate our findings, explore the technical aspects of this ultrasound methodology, and assess its potential role in predicting and preventing cognitive and postoperative complications.
