# Supplementary material for: Femoral Vein Pulsatility and Neurocognitive Disorder in Cardiac Surgery
Source: CJC Open. 2024 Nov 8;7(2):187–92. doi: 10.1016/j.cjco.2024.11.002 (PMC11886367; doi:10.1016/j.cjco.2024.11.002)
Supplement: Supplementary Data [file mmc1.pdf]

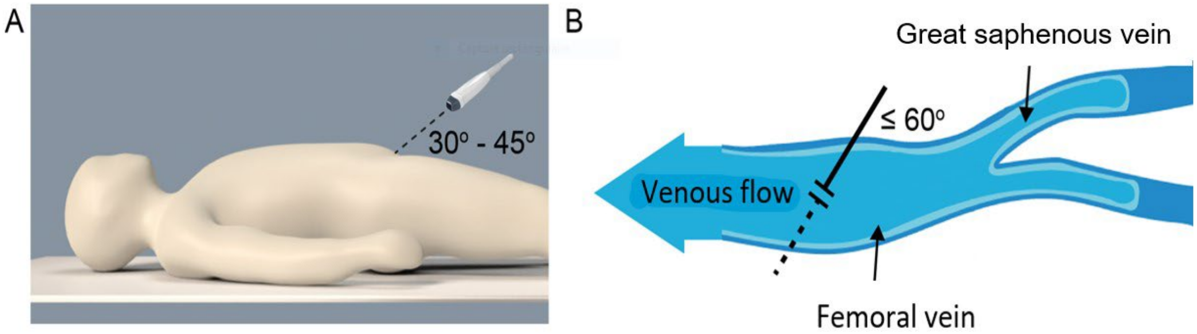

Supplemental Figure S1. How to perform the Doppler examination of the femoral vein.[1-3]

Patient's position (A), ultrasound probe position (A), and cursor position in relation to venous flow (B).

In order to examine the veins of the lower limbs, the bed should be in a horizontal position with the head inclined at 20° or less. To facilitate venous filling of the lower limbs and allow some dilation of the veins, the reverse Trendelenburg position can also be used. For optimal exposure of the great saphenous vein, a slight flexion of the knee and external rotation of the hip can be performed.

Examination of the femoral vein (FV) begins from the inguinal ligament, and it's preferable to start with its proximal part since it is of larger caliber and easier to detect.

Doppler examination can be obtained by examining the FV in its short axis (transverse view) or in its long axis (longitudinal view), using a high-frequency linear probe. In the short axis, the image is easy to acquire and allows for a rapid qualitative assessment of the waveform produced. The long axis view has the advantage of allowing reliable quantification of observed venous flow velocities by correcting the angle of the cursor to less than 60° to be in line with the vessel and not underestimate the Doppler signal.

The velocity scale of the color signal should initially be set at 10 cm/s and then adjusted as needed. During the short-axis view, the probe should be tilted cephalically up to 45° to observe the venous flow normally moving away from the probe. Color Doppler is used to estimate velocity. The spectral Doppler profile is obtained by positioning the cursor in the middle of the vein and using

Pulse Wave Doppler (PWD). For quantitative evaluation of velocities, the spectral Doppler profile should be performed in a long-axis view with angle correction to less than 60°. Slight pressure should be applied to avoid vein compression.

The Doppler of the FV is normally non-pulsatile and fluctuates with respiration. When it is pulsatile, the FV Doppler waveform adopts a configuration similar to that of hepatic veins with **S** (systole) and **D** (diastole) waves, as well as an **a** wave and a **v** wave. The terms "antegrade" and "retrograde" are used to describe the direction of a wave, depending on whether it is in the same direction or opposed to the blood flow. The **S** and **D** waves are antegrade, the **a** wave may be retrograde, and the **v** wave can be either neutral, antegrade, or retrograde.

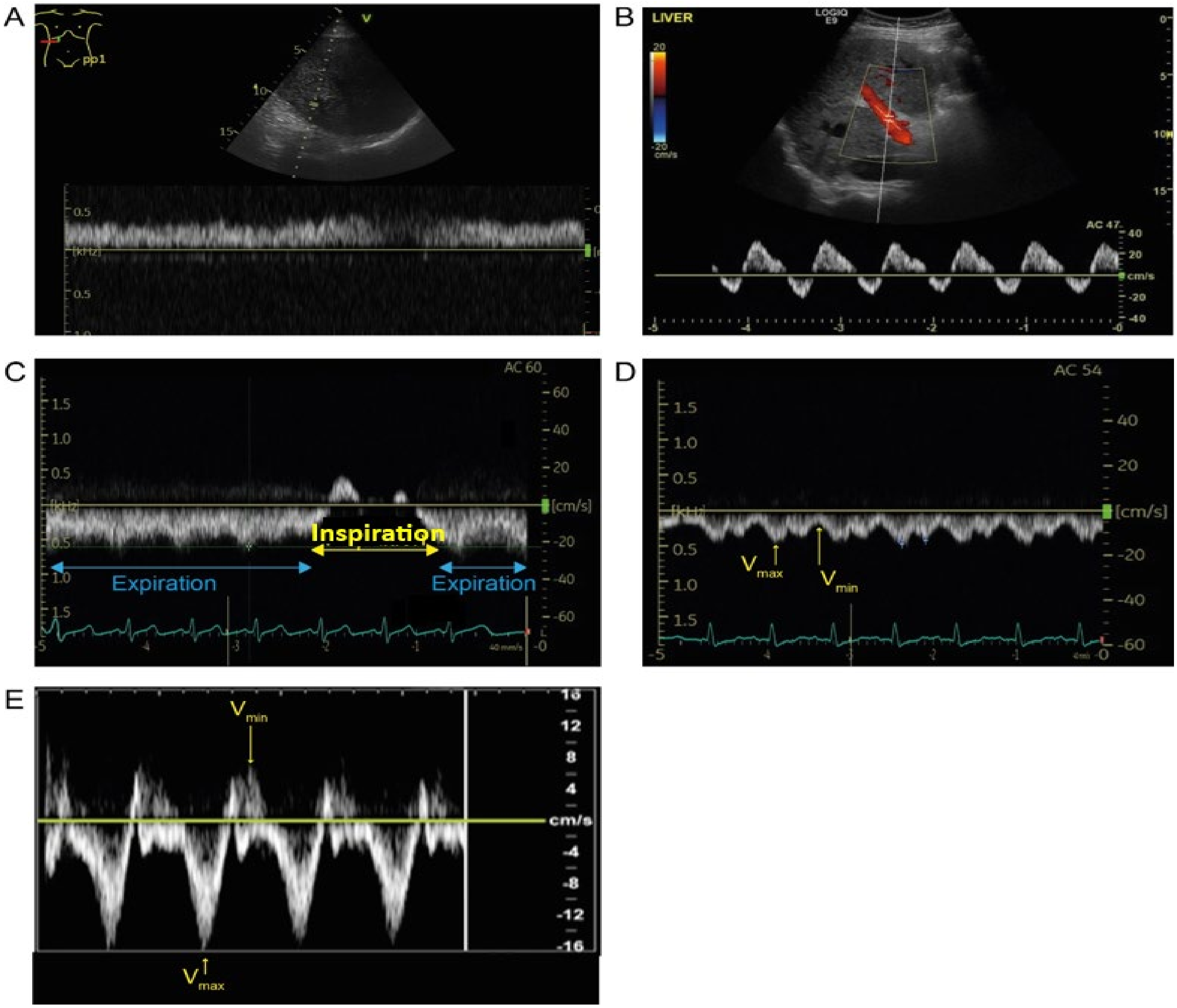

Supplemental Figure S2. Representation of Doppler ultrasound of the femoral vein (FV) and normal and pathological portal vein.

(A) Monophasic non-pulsatile portal vein; (B) Pulsatile and Biphasic Portal Vein; (C) FV, Disappearance of Doppler signal with respiration (respiratory phasicity); (D) FV, Pulsatile Doppler signal alternating with cardiac beats (cardiac modulation); (E) FV, reversed Doppler signal (retrograde) compatible with flow opposite to the normal direction of venous flow.

V max, maximum velocity; V min, minimum velocity.

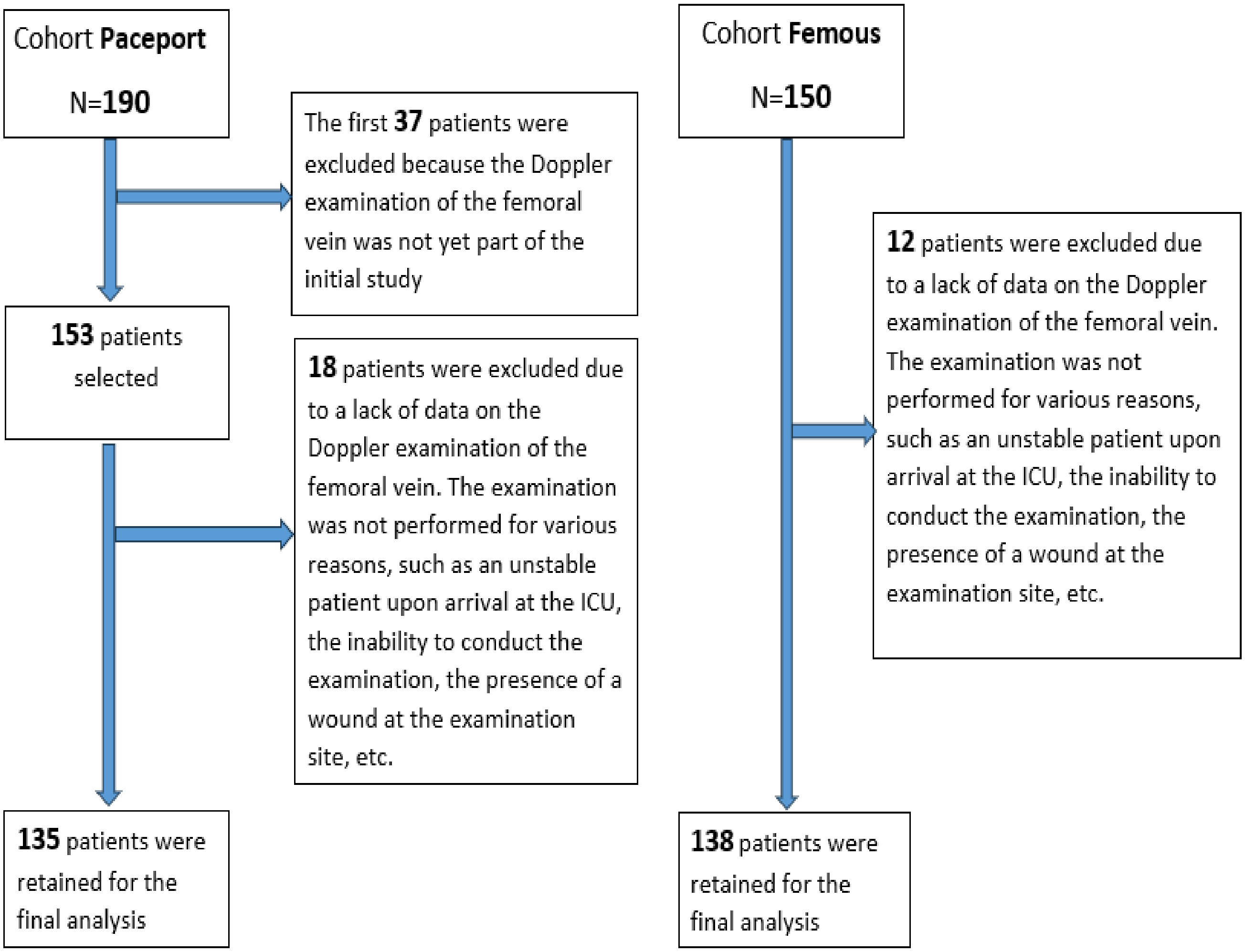

Supplemental Figure S3. Diagram of patient's selection.

Supplemental Table S1. Description and definitions of variables

Variables	Description and definition
**Demographic data and clinical characteristics**	
**Age**	All adults aged 18 and above. Calculated in years.
**BMI**	Body mass index. Expressed in kg/m^2.
**Alcohol use**	Alcohol consumption documented in the medical record or reported by the patient.
**Smoking**	Tobacco consumption documented in the medical record or reported by the patient.
**Diabetes**	Insulin-dependent or non-insulin-dependent diabetic patients.
**Chronic lung disease**	Chronic lung disease documented in the medical char.t
**EuroSCORE II**	Preoperative risk scores for adult cardiac surgery.[4]
**LV function**	Left Ventricular Function:<br>1 - Good (Left Ventricular Ejection Fraction (LVEF) > 50%),<br>2 - Moderate (LVEF = 31% - 50%),<br>3 - Poor (LVEF = 21% - 30%),<br>4 - Severe (LVEF < 20%).
**Echographic data**	
**FV pulsatility (index)**	Index of pulsatility of the FV (%), calculated according to the following formula: [(max velocity) - (min velocity)]/Max velocity ($\frac{Vmax-Vmin}{Vmax}$). Velocity is expressed in cm/s.
**Intraoperative data**	
**Cardiopulmonary bypass duration (CPB)**	Total duration of cardiopulmonary bypass in minutes until decannulation.
**CPB separation classification**	Weaning from cardiopulmonary bypass. Classified into 3 categories:[5,6]<br>1 - Easy (with one of the following: no medication or a single class of IV vasopressors);

Variables	Description and definition
	2 - Difficult: At least two of the following elements: IV inotropes, IV vasopressors, or inhaled pulmonary vasodilators (nitric oxide, milrinone, or epoprostenol);<br>3 - Complex: Failure of the first weaning attempt or mechanical circulatory support required.
**Postoperative Complications**	
**Intubation duration**	Duration of intubation from arrival in the intensive care unit until extubation. Calculated in hours up to 28 days postoperatively. Maximum value = 672 hours (24 hours/day * 28 days).
**Vasopressors use**	Total duration of vasopressor uses in the postoperative period. The following medications are considered: norepinephrine, phenylephrine, vasopressin, and/or inotropes (milrinone, dopamine, dobutamine, and adrenaline). Calculated in hours up to 28 days postoperatively. Maximum value = 672 hours (24 hours/day * 28 days).
**Time of persistent organ dysfunction (TPOD)[7]**	Duration of organ support: The cumulative time with support to keep one or more organs alive, in other words, TPOD represents the total time with this support or death during the first 28 days after surgery. This support includes mechanical ventilation; vasopressor therapy (continuous need for vasopressor agents such as norepinephrine, epinephrine, vasopressin, dopamine > 5 µg/kg/min, or phenylephrine > 50 µg/min); mechanical circulatory support (continuous need for mechanical devices such as an intra-aortic balloon pump or ECMO); and continuous or intermittent acute dialysis. Participants who died during the observation period had TPOD imputed from the time of death until the end of the observation period (24 hours for 28 days = 672 hours).

Variables	Description and definition
**Intensive care unit (ICU) length of stay**	Duration of stay in the ICU, from admission to discharge (including readmission to the ICU during the study period). Calculated in hours up to 28 days postoperatively. Maximum value = 672 hours (24 hours/day * 28 days).
**Hospital length of stay**	Total duration of hospitalization postoperatively (days). Calculated in days up to 28 days postoperatively. Maximum value 28 days.
**Acute kidney injury (AKI)**	AKI, defined according to: Improving Global Outcomes (KDIGO) based on the level of creatinine increase compared to the baseline preoperative value.[8]<br>Stage 1 : creatinine increase ≥ 27 µmol/L in 48 h or ≥ 50% in 7 days after surgery,<br>Stage2 : ≥ 100% increase in creatinine,<br>Stage 3 : ≥ 200% increase in creatinine or creatinine value exceeding ≥ 354 µmol/L with a minimum increase of 27 µmol/L from the baseline value, or initiation of dialysis.
**Major bleeding**	Major bleeding, calculated according to Bleeding Academic Research Consortium (BARC)[9] criteria, based on the following elements: intraoperative intracranial hemorrhage within 48 hours postoperatively, or re-intervention after sternotomy closure to control bleeding, or transfusion of ≥ 5 units of whole blood red cell concentrate within a 48-hour period, or chest tube drainage ≥ 2 L within a 24-hour period.
**Surgical reoperation**	Surgical reintervention for major bleeding or complications related to surgery.
**Stroke**	Defined by a persistent central neurological deficit lasting more than 72 hours.[10]

Supplemental References

1. Denault AY, Aldred MP, Hammoud A, et al. Doppler Interrogation of the Femoral Vein in the Critically Ill Patient: The Fastest Potential Acoustic Window to Diagnose Right Ventricular Dysfunction? Crit Care Explor 2020;2(10):e0209. doi:10.1097/cce.0000000000000209

2. Pellerito JS, Polak JF. Introduction to Vascular Ultrasonography. 6th Ed. Elsevier Saunders 2012:653.

3. Lee DK, Ahn KS, Kang CH, Cho SB. Ultrasonography of the lower extremity veins: anatomy and basic approach. Ultrasonography (Seoul, Korea) 2017;36(2):120-30. doi:10.14366/usg.17001

4. Nashef SA, Roques F, Sharples LD, Nilsson J, Smith C, Goldstone AR, Lockowandt U. EuroSCORE II. Eur J Cardiothorac Surg 2012;41(4):734-44. doi:10.1093/ejcts/ezs043

5. Denault AY, Tardif JC, Mazer CD, Lambert J; BART Investigators. Difficult and complex separation from cardiopulmonary bypass in high-risk cardiac surgical patients: a multicenter study. J Cardiothorac Vasc Anesth 2012;26(4):608-16. doi:10.1053/j.jvca.2012.03.031

6. Denault AY, Bussieres JS, Arellano R, et al. A multicentre randomized-controlled trial of inhaled milrinone in high-risk cardiac surgical patients. Can J Anesth 2016;63(10):1140-53. doi:10.1007/s12630-016-0709-8

7. Stoppe C, McDonald B, Benstoem C, et al. Evaluation of Persistent Organ Dysfunction Plus Death As a Novel Composite Outcome in Cardiac Surgical Patients. J Cardiothorac Vasc Anesth 2016;30(1):30-8. doi:10.1053/j.jvca.2015.07.035

8. Kidney Disease: Improving Global Outcomes (KDIGO) Acute Kidney Injury Work Group. KDIGO Clinical Practice Guideline for Acute Kidney Injury. Kidney Inter Suppl 2012;2:1-138.

9. Mehran R, Rao SV, Bhatt DL, et al. Standardized Bleeding Definitions for Cardiovascular Clinical Trials. Circulation 2011;123(23):2736-47. doi:10.1161/circulationaha.110.009449

10. Sun LY, Chung AM, Farkouh ME, van Diepen S, Weinberger J, Bourke M, Ruel M. Defining an Intraoperative Hypotension Threshold in Association with Stroke in Cardiac Surgery. Anesthesiology 2018;129(3):440-7. doi:10.1097/aln.0000000000002298
